# The President’s Address

**Published:** 1902-09

**Authors:** 


### THE PRESIDENT'S ADDRESS.

IT has been many years since an address by its chief officer has been so salient with suggestions and recommendations as the one presented to the American Veterinary Medical Association at Minneapolis. An effort was made during the sessions of the Convention to have it especially referred to the Executive Committee for consideration, but that body was so busy wasting its own and the Convention's time over some legal technicalities (in which field they have no status or place) over a worse than worthless member that they could not find time to take up these matters seriously.

While we cannot fully agree with all the recommendations of the President, we are in hearty accord with his views on many important questions so ably discussed in his excellent address, and we sincerely trust that the newly appointed Executive Board will find them worthy of a special session of that body, and not be allowed to slumber until September, 1903, when they must of necessity go over another year for execution. We trust that another year this body will not be affected with Morrisphobia that so completely caused a condition of innocuous desuetude to hang as a pall over its proper sphere of work for 1902.

